# Pathogenicity of avian reovirus variant in the immune organs of broiler chicks

**DOI:** 10.1016/j.virusres.2025.199538

**Published:** 2025-02-06

**Authors:** Haiyang Yu, Yudong Zhu, Qiong Wu, Wanjun Zhao, You Wang, Dong Wang, Huaguang Lu, Youxiang Diao, Yijing Li, Yi Tang

**Affiliations:** a College of Veterinary Medicine, Northeast Agricultural University, Harbin, China; b Tianjin Ringpu Bio-Technology Co, Ltd., Tianjin, China; c College of Animal Science and Technology, Shandong Agricultural University, Taian, China; d Department of Veterinary and Biomedical Sciences, The Pennsylvania State University, University Park, PA, 16802, USA; e Mount Taishan Shengliyuan Group Co, Ltd., Taian, China; f Institute of Animal Science, Chinese Academy of Agricultural Sciences, Beijing, China

**Keywords:** Avian reovirus, Immune organs, Suppressive infection, Chickens

## Abstract

In recent years, the infection rate of avian reovirus (ARV) in chicks has been continuously increasing, seriously endangering the healthy development of China's chicken farming industry. However, the pathogenicity of this virus in immune organs has been less studied. In this study, an ARV strain (Chicken/XJ/China/2022/01/16) was isolated from arthritic broiler chicks in Xinjiang province and whole genome sequencing of the ARV strain was performed using next generation sequencing (NGS) technology, and sequence analysis showed that the strain was a type V variant of the ARV gene. In order to further understand the pathogenicity of this strain in terms of immune organs, we randomized 120 healthy 1-day-old broilers equally into 3 groups (oral, footpad inoculation and control), where 2 experimental groups were inoculated with 0.2 mL of ARV (TCID_50_ of 10^5.5^ /0.1 mL) and the control group was inoculated with an equal amount of sterile saline. The results showed that the footpad inoculation group showed more severe symptoms, which, in addition to causing chick dwarf syndrome and viral arthritis, would also cause serious effects on the immune organs of the chicks. Among them, the spleen and bursa were most seriously damaged, the symptoms of arthritis appeared relatively late, which indicated that the spleen and bursa might be the main target organs preferentially attacked by avian reovirus. This study provides a rationale for the prevention and control of emerging avian reovirus and adds to the evidence of the effects of ARV on immune organs, with the expectation that it will be useful in understanding the immune response-related aspects of the disease in subsequent studies.

## Highlights

• By conducting genome-wide analysis of the isolated ARV variant strains, we further understood the patterns of ARV variation.
• Our research results have enriched the research on the pathogenicity of avian reovirus (ARV) in immune organs.
• Our research results have enriched the study on the pathogenic mechanisms of avian reovirus (ARV) variants, with the expectation of providing a theoretical basis for the subsequent prevention and control of reovirus.

## 1 Introduction

Avian reovirus (ARV) is member of the Orthoreovirus genus (Labrada et al., 2002). It's icosahedral non-enveloped double-stranded RNA (dsRNA) viruses with a particle size of 70–80 nm containing ten genome segments (Spandidos and Graham, 1976). The linear genome segments were molecularly divided into three size classes (L, M and S) based on their electrophoretic mobility on a polyacrylamide gel (Spandidos and Graham, 1976; Varela and Benavente, 1994). The ARV genome encodes four non-structural proteins (μNS, μNS, P10 and P17) and eight structural proteins (λA, λB, λC, μA, μBC, σA, σB and σC) (Bodelón et al., 2001; Schnitzer et al., 1982; Mor et al., 2014). The σC protein is expressed by the third open reading frame of the S1 gene. This protein is 326 amino acids long and is the most prone to mutation in the genome of the reovirus (Calvo et al., 2005). σC is able to bind to host cell surface-specific receptors and is a major immunogenic surface protein containing type and a wide range of specific neutralizing epitopes (Ayalew et al., 2017). Notably, it is the most divergent among reovirus proteins. The σC protein has thus become an important factorin the identification of ARV genotype, analysis of sequence variation, studies of immunogenic relationships, and cross-protection between different strains (Jiang et al., 2023; Bodelón et al., 2001).

ARV can infect a wide range of avian species and cause a variety of diseases, and has been isolated from many birds in nature, including chickens, turkeys, geese, ducks, pigeons, and quail, but broilers and broiler breeders are the most clinically affected (Jones, 2000). It is characterized by depression, lameness, swollen foot pads, arthritis, tenosynovitis, slow growth and decreased immune function. Autopsy reveals synovial and articular swelling, joint effusion, and in some cases hemorrhagic spots in the duodenum, synovium, and joints, and ulceration of the tarsal cartilage. Since 2015, the emergence of ARV variants has led to increasing losses due to ARV infection.

In addition, immune organs such as spleen, liver and bursa of Fasciola were found to be the site of initial viral proliferation and an important part of the ARV infection pathway by Tang et al. (1987). This study was based on the gene type V Avian Reovirus (ARV) strain, and systematically investigated the latent infection period, virus shedding pattern, and damage to major immune organs of the virus through methods such as real-time fluorescence quantitative PCR, indirect ELISA, and immunohistochemistry. It is hoped that this study will be helpful for understanding the immune responses related to subsequent research on this disease.

## 2 Materials and methods

### 2.1 Ethics statement

All the animal infection experiments were approved by the Animal Care and Use Committee of Shandong Agricultural University and conducted in accordance with the “Guidelines for Experimental Animals” of the Ministry of Science and Technology (Beijing, China).

### 2.2 Virus

The ARV isolate (Chicken/XJ/China/2022/01/16) used in this study was obtained from a diagnostic case of a submitted samples of tendons and joint fluid from broilers with arthritis symptoms in Xinjiang Province, China. Tendon and joint fluid samples were collected, ground to a slurry in sterile DMEM, sterilized by repeated freeze-thawing and filtration through a 0.22μm filter cell, and the suspension was inoculated into chicken liver cancer (LMH) cells. Viral stocks were harvested when virus-infected 75% cells exhibited cytopathic effect (CPE).

The titer of Chicken/XJ/China/2022/01/16 in LMH cells was determined according to the half-tissue culture infectious dose (TCID_50_). LMH cells were seeded in 96-well plates, inoculated with 105 cells per well, and incubated for 48 h at 37 °C under 5% CO2. The viral solution was serially diluted 10-fold, and100μl was added to each well. The CPE was measured 4 days after inoculation and calculated by the Reed and Muench method (Reed and Muench, 1938).

### 2.3 Next-Generation sequencing and analysis

We extracted viral RNA from the Chicken/XJ/China/2022/01/16 infected cell culture using a Total Viral RNA Isolation Kit (Ambion) following the manu‐facturer's instructions. We used the TruSeq RNA Sample Preparation Kit (illumina) through the manufacturer's instructions to process the total RNA samples from to prepare sequencing libraries. We purified the library fragment using the AMPure XP system (Beckman Coulter) in length and the prepared cDNA library was loaded on the MiSeq sequencer to get the raw NGS reads. All NGS raw data were analyzed and reassembled by different modules of QIAGEN software. Virus (ORF) prediction, nucleotide (nt) and amino acid (aa) sequence comparison analyses were performed using the Editseq and Megalign modules of DNASTAR 7.1 (DNASTAR, Inc.) (Yang et al., 2018). The BLAST program on the NCBI website was used to search for published related isolates and further analyze homology relationships (Table 1). We constructed phylogenetic trees of all gene segments with MEGA V7.0 program using the neighbour‐join‐ing method and a bootstrap value selected for 1000 replications (Teng et al., 2014; Tang and Lu, 2015).

**Table 1:** Accession number of the reference strain used.

Reference strains	S1133	1733	PA01224A	AVS-B	918	138	PA15511
λA	AY547458	KF741706	KT428304	FR694191	AY641738	EU707933	KP731611
λB	DQ534201	KF741707	KT428299	FR694192	–	EU707935	KP731612
λC	DQ300175	KF741708	KT428300	FR694193	AF301473	EU707937	KP737613
μA	DQ300176	KF741709	KT428301	FR694194	AY639617	AY557188	KP731614
μB	DQ300177	KF741710	KT428302	FR694195	AY635945	AY750052	KP731615
μNS	AY608700	KF741711	KT428303	FR694196	AY573911	AY557190	KP731616
σA	AF104311	KF741713	KT428305	FR694198	AF294766	AF059717	KP731618
σB	AO20642	KF741714	KT428306	FR694199	AF301473	AF059721	KP731619
σC	AF330703	KF741712	KT428304	FR694197	AF297215	AF218359	KP731617
σNS	AOU95952	KF741715	KT428307	FR694200	AF294775	AF059725	KP731620

### 2.4 Animal experiments

One hundred and twenty 1-day-old Ross 308 broilers were randomly divided into three groups of 40 broilers each, with Group A being the oral group, Group B being the footpad group, and Group C being the negative control group. 0.2 mL of ARV was administered orally to Group A at 1 day of age, 0.2 mL of saline was inoculated into the footpad of Group B at 1 day of age, and 0.2 mL of saline was administered orally to Group C at 1 day of age. Clinical symptoms were observed and recorded daily.

One hundred and twenty 1-day-old Ross 308 broilers were randomly divided into three groups of 40 birds each, with group A as the oral group, group B as the footpad group, and group C as the negative control group. The breeder flocks were vaccinated with S1133 weak vaccine on day 8 and week 8, respectively, and inactivated S1133 vaccine before laying. 0.2 mL of the Chicken/XJ/China/2022/01/16 virus (TCID50 =10^5.5^/0.1 mL) was administered orally to Group A at 1 day of age, 0.2 mL of the Chicken/XJ/China/2022/01/16 virus (TCID50 =10^5.5^/0.1 mL) was inoculated into the footpad of Group B at 1 day of age, and 0.2 mL of saline was administered orally to Group C at 1 day of age. Clinical symptoms were observed and recorded daily.

### 2.5 Sample collection

Three cloacal swabs were collected from chicks every 2 days after inoculation, and three anticoagulated blood samples were collected using sodium heparin-containing blood collection tubes until day 30 post-inoculation. Cloacal swabs were immersed in 1 mL of physiological saline, frozen and thawed twice, and then stored at −80 °C for RNA extraction and subsequent testing.

### 2.6 Analysis of immune organ indices

Three chicks from each group were randomly selected, weighed, euthanized and necropsied at 2-day intervals until 30th day. The gross pathological changes were observed, and immune organs such as bursa, liver, spleen, thymus, and cecum tonsils were taken for calculation of immune organ index (immune organ index = (weight of immune organs)/ (body weight of the chick) × 100%), determination of viral load, and pathological and immunohistochemical examinations.

### 2.7 Histopathological observations

A part of all organs were placed in 10% formalin phosphate buffer for organ fixation for 3∼5 d. The tissues were trimmed and blocked, and the histological sections for general pathology were prepared by paraffin embedding, dehydration, slicing and drying, and stained and sealed with hematoxylin and eosin (H&E) staining using standard methods. The histopathological changes of each organ were observed under a light microscope (Nikon, Eclipse E100).

The other part of all organs was immersed in 4% paraformaldehyde for organ fixation, and then the tissues were trimmed, fixed and embedded to make paraffin sections, which were deparaffinized and rehydrated by two xylene solutions, incubated with 3% H_2_O_2_ for 20 min at room temperature to block endogenous peroxidase, followed by antigenic restoration in citrate buffer for 40 min and incubated with 5% BSA at 37 °C for 40 min, primary antibody (laboratory prepared ARV monoclonal antibody, 1:1000) was incubated for 1 hour, and the secondary antibody (biotin-labeled goat anti-mouse IgG of Cowin Biotech Co., Ltd., 1:500) was incubated for 1 hour at 37 °C, and the color was developed by adding a drop of HRP-DAB chromatography solution, rinsed with tap water, stained with hematoxylin, dehydrated, transparent, and sealed. Finally, the histopathological changes of each organ were observed under light microscope (Nikon, Eclipse E100).

### 2.8 Genomic RNA extraction and quantitative real-time pcr

Total RNA was extracted from swabs and tissue samples using the MiniBEST Universal RNA Extraction Kit (TaKaRa) and the concentration of RNA samples was measured using a DeNovix DS-11 spectrophotometer (Nanodrop, Wilmington, DE) and stored at −80 °C. Viral load detection in the samples was performed using the TaqMan RT-qPCR method described in our previous study (Jiang et al., 2021a). The reaction mixture contained 10 μL of Premix Ex Taq (2x), 0.4 μL of forward primer, 0.4 μL of reverse primer, 0.4 μL of ROX reference dye, 6.4 μL of ddH2O, 0.4 μL of the probe, and 2.0 μL of DNA template. The reactions were conducted accordingto the following conditions: 42 °C for 5 min, 95 °C for 10 s, 40 cycles at 95 °C for 5 s, and 60 °C for 20 s. The ARV-speciffc primers and TaqMan probe were designed to target the M1 gene of ARV isolate (forward: 5′-ATGGCCTATCTAGC-CACACCTG-3′ and reverse: 5′- CAACGTGATAGCATCAATAGTAC-3′ and TaqMan probe: 5′-FAMCAACGTGATAGCATCAATAGTAC-BHQ1-3′).

### 2.9 Antibody level detection

Leave the anticoagulated blood specimens at a constant temperature of 37 °C for 30 mins. When a small portion of plasma precipitates from the upper layer, centrifuge the specimens at 1500 × *g* for 10 mins. Then carefully aspirate the upper serum layer and store it at −80 °C. Use the laboratory-prepared ARV indirect ELISA assay to conduct antibody detection on the collected serum. Take ARV σC-pET-32a recombinant protein (at a dilution ratio of 1:1000) as the antigen and coat it overnight at 4 °C. Incubate it with 5% skimmed milk powder at 37 °C for 2 h. Incubate the primary antibody (serum, at a dilution ratio of 1:10) at 37 °C for 1 hour. Use the rabbit anti-chicken IgG-HRP enzyme-labeled antibody (from Beyotime Biotechnology, at a dilution ratio of 1:1000) as the secondary antibody and incubate for 1 h. Develop the color with TMB chromogenic solution (from TIANGEN) at 37 °C for 15 mins. Use 2 M sulfuric acid as the stop solution to stop the reaction. Finally, measure the OD450 values of each well using an ELISA microplate reader (ELx800, Bio Tek) (Liu, 2019).

### 2.10 Statistical analysis

All results are expressed as mean ± standard deviation and were analyzed using the one-way analysis of variance (ANOVA) procedure of GraphPad Prism 6.0 (GraphPad Software Inc., San Diego, CA, USA). *P* < 0.05 was considered a statistically significant.

## 3 Result

### 3.1 Virus isolation and characterization

**Fig. 1:**
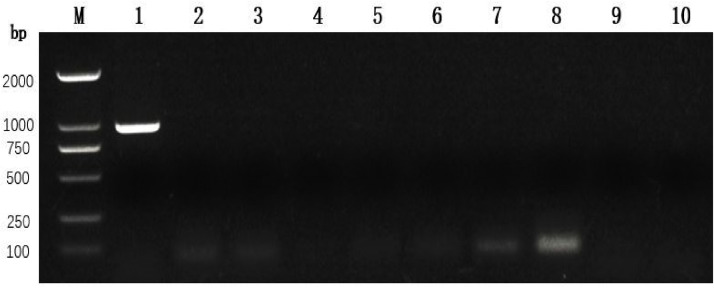
Purity testing. M. DNA marker(2000 bp); 1. ARV; 2. MS; 3. MDRV; 4. NDRV; 5. H9 AIV; 6. IBDV; 7. NDV; 8. FAV; 9. IBV; 10. Negative control.

The second-generation virus was inoculated into LMH cells in good culture condition for 36 h after the appearance of cell fusion and the formation of CPE with inclusion bodies, which was positive for ARV and negative for other common pathogens by RT-PCR. The results showed that we successfully isolated a strain of ARV virus and named it Chicken/XJ/China/2022/01/16. According to the method of Reed and Muench, the virus titer was determined as TCID50 =10^5.5^/0.1 mL. Virus purity assays showed positive ARV that viral cultures but negative results for common Chicken viruses (Inffuenza virus (H9), Mycoplasma synoviae, muscovy duck reovirus, novel duck reovirus, infectious bursal disease virus, newcastle disease virus, Fowl adenovirus, and Infectious bronchitis virus) (Fig. 1).

### 3.2 Phylogenetic analyses and sequence analysis

**Fig. 2:**
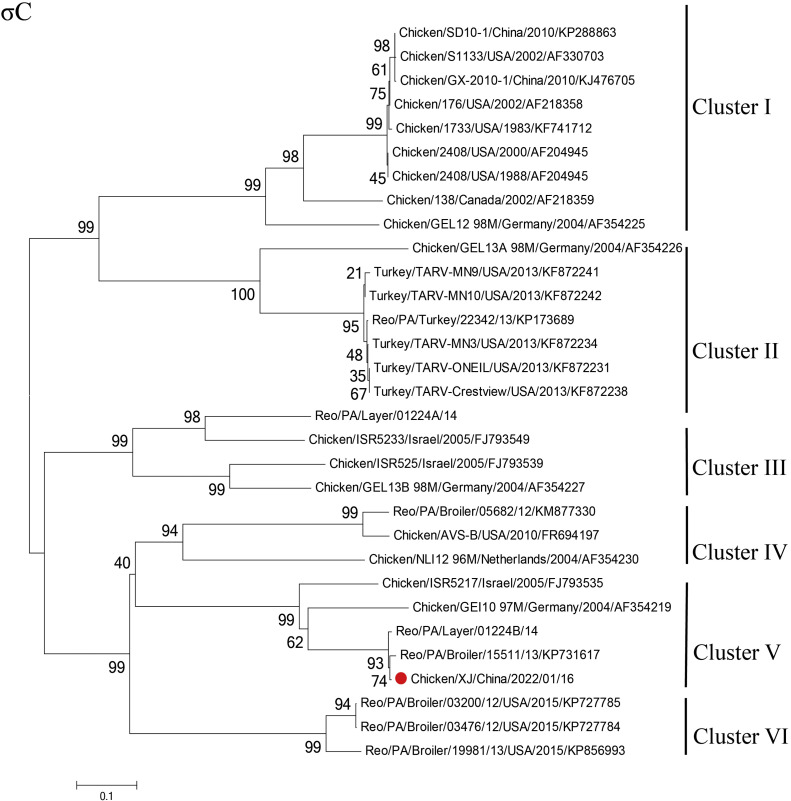
Phylogenetic trees constructed by avian orthoreovirus (ARV) based on nucleotide sequences of the σC gene fragments. Note: The 487 Chicken/XJ/China/2022/01/16strain was marked with a red-colour circle.

Genetic evolutionary analysis (Fig. 2) showed that Chicken/XJ/China/2022/01/16 strain (PP188008∼PP188017) is a close relative to the ARV gene type V variant PA15511. In addition, the λA, λB, uA, uB, uNS, σA and σB gene fragments of this isolate were genetically closer to the S1133 strain, and the λC and σNS gene fragments were genetically closer to the AVS-B strain.

The nucleotide (nt) and amino acid (aa) sequences of the coding regions of the Chicken/XJ/China/2022/01/16 strain showed the different sequence identity values with reference chicken-origin reovirus strains, including historical and pathogenic strains (Table 1), and the results of pairwise comparisons of nt and aa are summarized in Table 2. In case of λA (95.3%∼95.6 nt;98.7%∼99.0% aa) λB (91.5%∼91.9% nt;98.0%∼98.5% aa) uA (95.1%∼95.4% nt;98.9%∼99.0% aa) uB (91.2%∼91.4% nt;94.7%∼94.8% aa) uNS (91.7% nt;95.8%∼95.9% aa) σA (96.2%∼96.8% nt;96.4%∼97.8% aa) σB (93.9%∼94.1% nt;97.6%∼98.1% aa) the calculations demonstrated the higher identity values with S1133, 1733 and SD10–1. However, λC (87.1% nt; 92.6% aa) showed high homology to PA15511 strain, σNS (89.3% nt; 96.2% aa) showed high homology to AVS-B strain. Sequence analysis demonstrated that Chicken/XJ/China/2022/01/16 strain might have acquired its current genomic composition through several homologous. In addition, the Chicken/XJ/China/2022/01/16 strain shared lower identity with conventional vaccine strains (S1133, 1733) in σC-encoding gene (56.3%∼56.7% nt,49.5%∼49.8% aa), which may be one of the reasons why the viral arthritis cases in broiler flocks possessed high level of maternal antibody has been increasing in China.

**Table 2:** Sequence identities between chicken-origin avian orthoreovirus (ARV) strain (Chicken/XJ/China/2022/01/16) and members of Orthoreovirus genus.

	L1-λA		L2-λB		L3-λC		M1-μA		M2-μB		M3-μNS		S2-σA		S3-σB		S1-σC		S4-σNS	
Isolated strain	nt	aa	nt	aa	nt	aa	nt	aa	nt	aa	nt	aa	nt	aa	nt	aa	nt	aa	nt	aa
S1133 Cluster I	95.3	98.7	91.5	98.0	81.8	88.9	95.3	98.9	91.4	94.8	91.7	95.9	96.2	96.4	93.9	97.6	56.3	49.8	87.7	96.0
1733 Cluster I	95.6	99.0	91.9	98.5	82.1	88.8	95.4	99.0	91.4	94.8	91.7	95.8	96.6	97.4	94.1	98.1	56.7	49.5	88.0	96.0
PA01224A Cluster III	90.3	98.5	86.8	96.9	86.9	91.9	89.8	97.5	87.7	94.5	87.9	93.7	92.4	95.9	87.4	94.6	58.4	51.4	88.0	95.7
AVS-B Cluster IV	90.8	98.8	78.9	94.6	86.3	92.5	90.3	98.2	77.8	92.3	87.4	93.9	92.6	95.7	88.6	97.6	66.1	60.9	89.3	96.2
918 Cluster I	90.2	98.3	–	–	81.3	88.3	91.0	98.9	76.4	88.3	84.9	93.1	95.0	92.6	90.8	95.9	62.6	60.6	83.3	94.0
138 Cluster I	92.2	98.7	87.7	97.9	88.2	92.5	91.7	97.8	88.0	95.4	89.2	95.0	96.8	97.8	88.6	95.9	56.6	51.1	88.3	96.2
PA15511 Cluster V	90.1	98.6	88.9	97.6	87.1	92.6	89.0	96.9	88.3	95.0	87.5	94.7	92.1	94.7	88.0	95.9	98.7	99.1	81.2	95.9
SD10-1 Cluster I	95.4	99.0	91.6	98.0	81.8	88.3	95.1	99.0	91.2	94.7	91.7	95.9	96.6	97.4	93.9	97.8	56.3	50.2	88.0	96.0
Turkey-23342 Cluster II	85.7	97.7	85.5	96.1	83.1	88.7	91.8	97.1	90.6	94.5	90.1	94.7	91.6	95.0	74.8	79.1	56.7	51.7	86.7	94.3

Abbreviations: aa, amino acid sequence; nt, nucleotide sequence.

### 3.3 Clinical manifestation and body weight loss

**Fig. 3:**
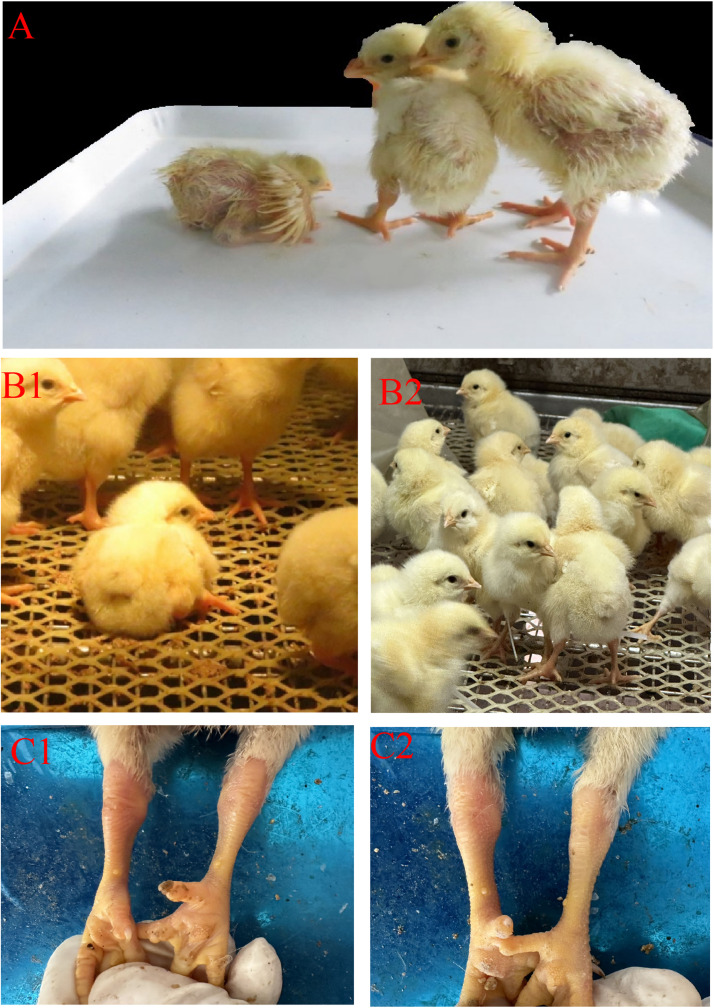
Clinical symptoms of infected chicks. (A) Most from left to right are footpad injection group, oral group and control group-10d. The footpad injection group had the most serious inhibitory effect on body weight, and the body weight of the oral group was slightly lower than that of the control group. (B1) Diseased ducks were paralyzed and unable to stand-8d. (B2) Control group was normal-8d. (C1) Swollen joints-10d. (C2) Normal joints-10d.

**Fig. 4:**
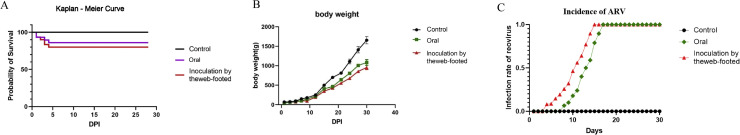
Survival curves, weight changes of infected chicks, and the incidence rate of Avian Reovirus. (ARV) in chicks. (A) Kaplan-Meier curves. (B) Weight change curves. (C) incidence of Avian Reovirus (ARV).

The pathogenicity of the isolate chicken/XJ/China/2022/01/16 to Ross 308 broiler chickens was studied using a 30-day clinical symptom monitoring method. After chicks were infected with ARV, different injection methods led to similar clinical symptoms, manifested as arthritis and growth retardation syndrome. Among them, the footpad injection group (Group B) was the most severely affected (Fig. 3). The statistical results of the incidence rate of chicks (Fig. 4C) showed that the footpad injection group (Group B) exhibited clinical symptoms earlier than the oral injection group (Group A). All chicks in both groups were diseased at 16 days of age, while no chicks in the control group (Group C) were diseased. The Kaplan - Meier curve of chicks (Fig. 4A) showed that no chicks in the control group (Group C) died. The deaths of chicks in the oral injection group (Group A) and the footpad injection group (Group B) were concentrated within 5 days post - infection (dpi). The mortality rate of Group B was higher than that of Group A, and the death situation was more severe. The weight detection results (Fig. 4B) showed that the broiler chicks gained weight more slowly after artificial infection, and the weight gain rate decreased significantly after 12 days post-infection (dpi). Compared with the control group, the body weight was 10% to 34% less, and this was particularly evident in Group B.

### 3.4 Pathologic examination and indexing of immune organs

Three broilers from the experimental and the negative control groups were randomly euthanized and drawn for necropsy evaluation, respectively. It was observed that the different modes of inoculation induced similar pathological changes, all causing greater damage to the immune organs, with more severe pathological changes in group B (Fig. 6). Enlarged and hemorrhagic thymus was seen at 5, 7, and 12 dpi, enlarged and hemorrhagic liver and spleen at 7, 15, and 21 dpi, and brittle bones of the leg at 12 dpi, with mucus and reddish ooze in the joint cavities, control group did not show pathological changes.

**Fig. 5:**
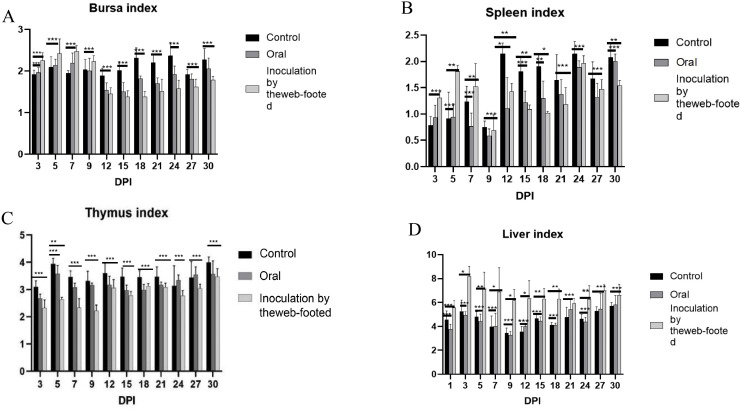
Immune organ indices of infected chicks. (A) Fasciola index (mg/*g* × 100%). (B) Splenic index (mg/*g* × 100%). (C) Thymus index (mg/*g* × 100%). (D) Liver index (mg/*g* × 100%).

After artificially infecting the broiler chicks, the changes in the immune organ indices of the chicks were counted (Fig. 5). The results showed that the 3–9 dpi bursa organ index was higher in the infected group than in the control group; the 3–7 dpi spleen organ index was higher than in the control group; the liver organ indexes in group B were all higher than in the control group, with 1–9 dpi being the most significant, and the thymus organ indexes and the liver organ indexes of group A did not change significantly in comparison with those of the control group.

**Fig. 6:**
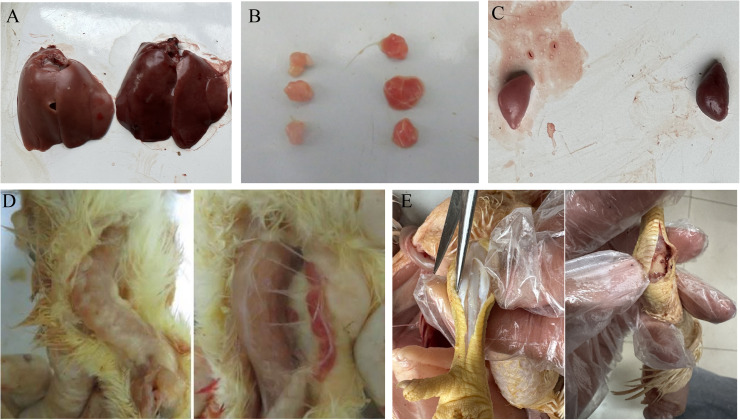
Changes in the dissection of different tissues and organs. Note: Control on the left, infected group on the right. (A) Bleeding from an enlarged liver-15d. (B) Bleeding from an enlarged thymus gland-15d. (C) Bleeding from an enlarged spleen 15d. (D) Apparent hemorrhage in the thymus gland-15d. (E) Significant bleeding in the joint cavity-21d.

### 3.5 Histopathologic analysis

**Fig. 7:**
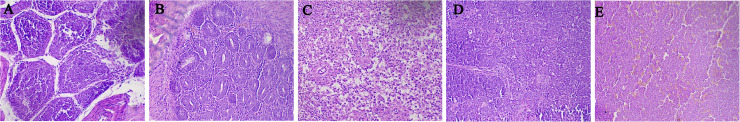
Histopathological changes in different organs-21d. (A) Interstitial edema of bursa. (B) Apoptosis of tonsillar lymphocytes. (C) Splenic lymphocyte disintegration. 509 (D) Thymic lymphocyte shedding. (E) Hepatic blood cell infiltration.

The histopathological results showed that there were basically no pathological changes in Group A. Chicks in group B showed bursal hemorrhage at 5 dpi, disintegration of lymphocytes in cecum tonsils with hemocyte infiltration, disintegration of splenic lymphocytes with apoptosis at 7 dpi, disintegration of thymus lymphocytes at 9 dpi, and disorganization of the hepatic cords with widening of sinusoidal gaps and hemocyte infiltration in the chicks at 12 dpi (Fig. 7).

### 3.6 Distribution of ARVs in immune organs

**Fig. 8:**
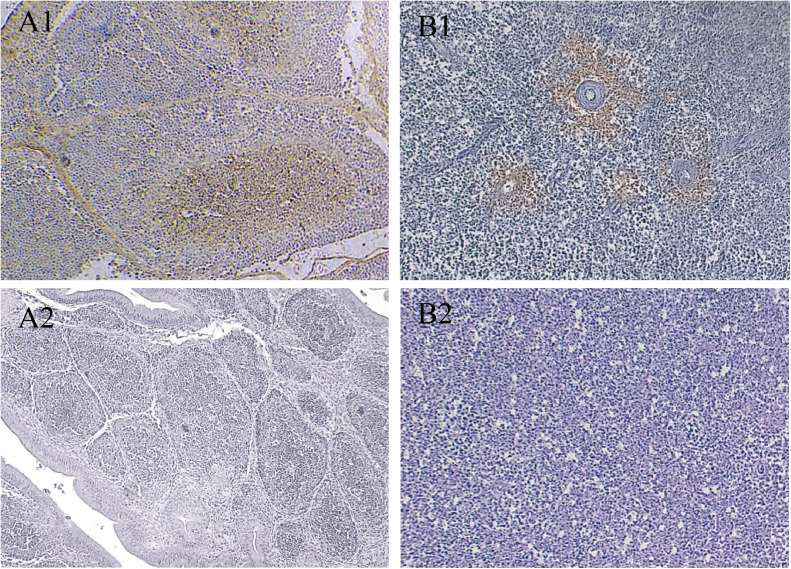
Immunohistochemical testing of more severely damaged immune organs. (A1) The bursa of Fabricius appeared to have a more intense positive signal. (B1) The spleen showed a stronger positive signal. (A2 and B2) corresponding tissue control.

Immunohistochemistry of broiler chicks artificially infected with ARV showed that positive signals were detected in the immune organs of the infected groups, and the spleens at 3 and 5 dpi and the bursa at 7 dpi were more severely damaged in group B, showing stronger positive signals (Fig. 8) Group A mainly appeared in the early stage of viral attack (1∼9 dpi), and the bursa and the spleen were both detected positively at 1 dpi, and the thymus was detected at 3 dpi (Table 3). signals were detected at 1 dpi in both the bursa and spleen, at 3 dpi in the thymus, and at 5 dpi in the liver (Table 3). Among them, the intensity of staining was higher at 3∼5 dpi, and the staining intensity of the bursa and spleen was higher than that of other immune organs. The damage in group B was more serious, and positive signals were detected in the bursa, spleen and thymus at 1 dpi, and in the liver at 3 dpi (Table 3). Among them, the staining intensity was higher at 3∼9 dpi, and the staining intensity of the bursa and spleen was higher than that of other immune organs.

**Table 3:** Dynamic distribution of positive signals in immune organs in A group.

Organ	Days Post Inoculation (DPI)											
	1	3	5	7	9	12	15	18	21	24	27	30
Bursa of Fabricius	+	++	+	+	+	+	+	–	–	–	–	–
Spleen	+	++	++	+	+	+	+	+	+	–	–	–
Thymus	–	+	+	+	+	+	+	–	+	–	–	–
Liver	–	–	+	–	+	+	–	+	–	+	–	–
Bursa of Fabricius	+	++	++	+++	+	+	+	+	+	+	–	–
Spleen	+	+++	+++	++	+	+	+	+	+	+	+	–
Thymus	+	+	++	+	+	+	+	–	+	–	–	–
Liver	–	+	+	++	++	+	+	+	+	+	–	+

*Abbreviations:* "-" indicates no positive signal, "+" indicates the presence of a positive signal, and "++" indicates a strong positive signal. Black for Group A; red for Group B.

### 3.7 Real time PCR analysis

**Fig. 9:**
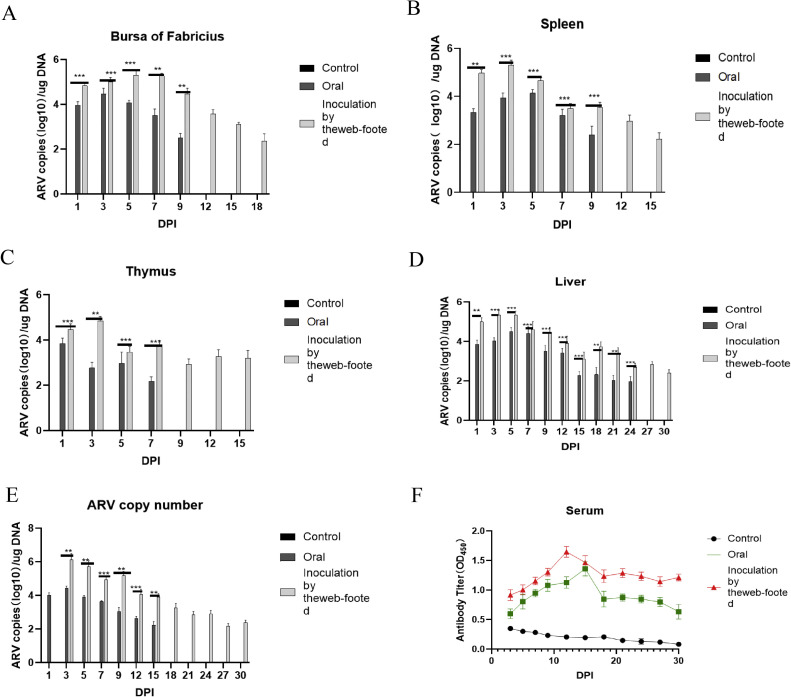
Tissue viral content and antibody levels. The data patterns for the two different inoculation methods are indicated using different colors. (A–D) Determination of virus content in the bursa of Fabricius, spleen, thymus and liver. (E) determination of virus content in cloacal swab samples. (F) results of the ELISA test for ARV antibody levels in serum.

The results of cloacal swab virus content in chicks at different time points after attacking by TaqMan probe (Fig. 9E) showed that group A started to detoxify at 1 dpi, and stopped detoxification after 15 dpi; group B started to detoxify at 3 dpi and continued to carry the virus. both groups A and B reached the peak of detoxification at 3 dpi, and the amount of detoxification showed a decreasing trend, but there were two small rises in group B at 9 and 24 dpi.

Detection of viral loads in immune organs at different time points using TaqMan probes showed that ARV copy numbers were similar in different immune organs, but viral levels and their variations were not similar between injection modalities (Fig. 9A∼D). The virus was detected at 1 dpi in all immune organs of the infected group, and the viral copy number and duration of viral load in group B were higher than those in group A. In group A, the peak was reached at 1 dpi in the thymus, 3 dpi in the bursa of the Fabricius and the liver and 12 dpi in the spleen; and in group B, the peak was reached at 3 dpi in the liver, the spleen, and the thymus, and at 5 dpi in the bursa of the Fabricius.

### 3.8 Serum antibody level testing

After artificially infecting the chicks, the changes in antibody levels in the serum showed (Fig. 9F) that the ARV antibody levels in the two experimental groups were higher than those in the control group. Among them, the ARV antibody level in the footpad injection group was higher than that in the oral injection group. The ARV antibody level in the oral injection group was the highest at 15 days post - infection (dpi), and the ARV antibody level in the footpad injection group was the highest at 12 dpi. Subsequently, the ARV antibody levels gradually decreased.

## 4 Discussion

ARV was first reported by Crawley and Fahey in 1954, and since then there have been increasing reports of ARV infections around the world (Jones et al., 1981; Meulemans et al., 1983; Tantawi et al., 1984). The disease had been effectively controlled by the application of commercialized avian reovirus vaccine, which solved the thorny problem of ARV transmission, but in recent years, there are more and more reports of infections by ARV in our country, and the sick chickens show depression, reduced appetite, swollen joints and paralysis. In the present study, genetic characterization of 10 segments and detailed pathogenicity test data confirmed that Chicken/XJ/China/2022/01/16 isolated from the above case was a V variant of the ARV gene.

Genetic evolutionary analyses suggest that Chicken/XJ/China/2022/01/16 may have undergone a recombination event. σC gene segments are less homologous to the conventional vaccine strains, strains S1133 and 1733, in terms of homology (56.3%∼56.7% nt,49.5%∼49.8% aa), and are more variable, which tends to result in immunization failures, which may be an important reason for the occurrence of ARV infection.

To further investigate the pathogenicity of the XJ strain on immune organs, we conducted an animal regression experiment using 1-day-old healthy Ross broilers. Broiler chicks artificially infected by different routes of inoculation showed depression, weight loss and arthritis, which were consistent with those observed in natural outbreaks, but the severity of the manifestations varied among the different routes of inoculation, with the footpad-infected group showing more severe clinical signs than the orally administered group. Damage to the immune organs by this strain of ARV occurred in the early stages of infection (1–9 dpi), and the condition of the chicks improved in the later stages, but their status could not be completely normalized. The results of body weight and symptoms showed that compared with the control group, the body weight of broiler chicks in the oral group was relatively low, and the mental state was slightly reduced, and some of them showed low appetite and thinness; there was a big difference in body size between broiler chicks in the footpad inoculation group and the control group, and the body weight of broiler chicks in the footpad inoculation group was reduced by about 800 g in 30 dpi, and there was a serious decline in the mental state in 24 h, and then they gradually showed symptoms such as poor growth, enlarged joints, and a fluctuating sensation when pressed, etc. Some of them lost normal walking. Some of the chicks lost normal walking ability, were lame and paralyzed. This indicates that infection of broilers with XJ strain results in swollen joints and developmental retardation, which is consistent with the results reported by RK Page (Page et al., 1982). The different pathological changes were similar in the infected group, but were more severe in the footpad inoculated group, with different degrees of tissue hemorrhage, hemocyte infiltration, interstitial edema, lymphocyte disintegration and loss, and widening of the interstitial space in the liver in all immune organs, which were similar to the results of the study by Troxler et al. (2013). Immunohistochemistry results showed that the staining intensity of the bursa and spleen in the infected group was higher than that of other immune organs, which indicated that the XJ strain mainly attacked the spleen and the bursa, leading to the dysfunction of the immune system and accelerating the invasion of other pathogens (Ni and Kemp, 1990). The results of viral load in immune organs showed that the virus appeared earlier and lasted longer in immune organs, and no more virus was detected in the spleen at 12 dpi, and the liver ended the latest, which may be related to its replication mechanism. Antibody levels showed that both test groups had higher ARV antibody levels than the control group. Among them, the ARV antibody level in the footpad injection group was higher than that in the oral injection group. ARV antibody levels peaked at 15 days post-infection (dpi) in the oral injection group and at 12 days post-infection (dpi) in the footpad injection group. This may be the result of the chicks' own immune response to the virus. The results of latent infection and detoxification showed that the chicks infected by oral administration had a longer latent infection time of about 9 d, even though their symptoms were relatively mild, and the chicks infected by footpad injection had obvious symptoms and a shorter latent infection time, but their detoxification time was long and could last for more than 30 d, which indicated that the different routes of infection would have a certain impact on the latent infection time of the disease, and that the widespread infection of ARV infection might be associated with the cloacal detoxification, which might be the result of the immune response to the virus. The widespread ARV infection may also be associated with cloacal detoxification. The results of immune organ index showed that the immune index of bursa and spleen in the oral group was higher than that of the control group in the early stage, and then gradually decreased and tended to be higher than that of the control group in the late stage, whereas the changes of thymus and liver were not significant, which might be related to the fact that ARV could not be replicated in all the tissues, and it also indicated that the ARV virus mainly occurred in the early stage of the infection. Therefore, we should uphold the principle of early prevention, early detection and early treatment, and we should also strengthen feeding management, take good disinfection measures and increase disinfection efforts to prevent the spread of pathogens.

In conclusion, the ARV genotype V variant strain XJ strain we isolated not only causes typical ARV symptoms associated with chick malaise, short body size and viral arthritis (Jiang et al., 2021b), but can also severely damage the immune organs of chicks. Among them, the spleen and the bursa were the most severely damaged and severely reduced the immunity of the chicks to cause secondary infections, while symptoms such as joints appeared relatively later, suggesting that the spleen and the bursa may be the main target organs preferentially attacked by ARV. Effective vaccination is important for the prevention and control of this virus, therefore, the development of vaccines that are effective against the current prevalent strains of ARV is a high priority. The present study is of great significance for the study of immunocompetence and immunosuppression in broiler chicks and may help in the prevention and control of ARV pathology.

## Author statement

The authors declare that the research was conducted in the absence of any commercial or financial relationship that could be construed as a potential conflict of interest.

## Funding

This work was supported by China Agriculture Research System (CARS-42-19) and Guangzhou Innovation and Entrepreneurship Leading Team Project (202009020009).

## CRediT authorship contribution statement

**Haiyang Yu:** Writing – original draft. **Yudong Zhu:** Formal analysis, Data curation. **Qiong Wu:** Software, Data curation. **Wanjun Zhao:** Visualization. **You Wang:** Validation, Software. **Dong Wang:** Validation. **Huaguang Lu:** Methodology, Conceptualization. **Youxiang Diao:** Resources, Methodology. **Yijing Li:** Supervision, Methodology. **Yi Tang:** Writing – review & editing, Supervision, Methodology, Conceptualization.

## Declaration of competing interest

The authors declare that the research was conducted in the absence of any commercial or financial relationship that could be construed as a potential conflict of interest.

## Data Availability

Data will be made available on request.

## Appendix Supplementary materials

Image, application 1

## References

[bib0001] Ayalew L.E., Gupta A., Fricke J., Ahmed K.A., Popowich S., Lockerbie B., Tikoo S.K., Ojkic D., Gomis S. (2017). Phenotypic, genotypic and antigenic characterization of emerging avian reoviruses isolated from clinical cases of arthritis in broilers in Saskatchewan, Canada. Sci. Rep..

[bib0002] Bodelón G., Labrada L., Martønez-Costas J., Benavente J. (2001). The avian reovirus genome segment S1 is a functionally tricistronic gene that expresses One structural and two nonstructural proteins in infected cells. Virology.

[bib0003] Calvo P.G., Fox G.C., Hermo Parrado X.L., Llamas-Saiz A.L., Costas C., Martínez-Costas J., Benavente J., Van Raaij M.J. (2005). Structure of the carboxy-terminal receptor-binding domain of avian reovirus fibre sigmaC. J. Mol. Biol..

[bib0004] Jiang X., Lin Y., Yang J., Wang H., Li C., Teng X., Tang Y., Diao Y. (2021). Genetic characterization and pathogenicity of a divergent broiler-origin orthoreovirus causing arthritis in China. Transbound. Emerg. Dis..

[bib0005] Jiang X., Lin Y., Yang J., Wang H., Li C., Teng X., Tang Y., Diao Y. (2021). Genetic characterization and pathogenicity of a divergent broiler-origin orthoreovirus causing arthritis in China. Transbound. Emerg. Dis..

[bib0006] Jiang X., Wei F., He D., Niu X., Wu B., Wu Q., Tang Y., Diao Y. (2023). Co-circulation of multiple genotypes of ARV in poultry in Anhui, China. Avian Pathol..

[bib0007] Jones R.C. (2000). Avian reovirus infections. OIE Rev. Sci. Tech..

[bib0008] Jones R.C., Guneratne J.R.M., Georgiou K. (1981). Isolation of viruses from outbreaks of suspected tenosynovitis (viral arthritis) in chickens. Res. Vet. Sci..

[bib0009] Liu H. (2019).

[bib0010] Labrada L., Bodelón G., Viñuela J., Benavente J. (2002). Avian reoviruses cause apoptosis in cultured cells: viral uncoating, but not viral gene expression, is required for apoptosis induction. J. Virol..

[bib0011] Meulemans G., Dekegel D., Charlier G., Froyman R., Van Tilburg J., Halen P. (1983). Isolation of orthoreoviruses from psittacine birds. J. Comp. Pathol..

[bib0012] Mor S.K., Sharafeldin T.A., Porter R.E., Goyal S.M. (2014). Molecular characterization of L class genome segments of a newly isolated turkey arthritis reovirus. Infect. Genet. Evol..

[bib0013] Ni Y., Kemp M.C. (1990). Selection of genome segments following coinfection of chicken fibroblasts with avian reoviruses. Virology.

[bib0014] Page R.K., Fletcher O.J., Rowland G.N., Gaudry D., Villegas P. (1982). Malabsorption syndrome in broiler chickens. Avian Dis..

[bib0015] Reed L.J., Muench H. (1938). A simple method of estimating fifty per cent endpoints. Am. J. Epidemiol..

[bib0016] Schnitzer T.J., Ramos T., Gouvea V. (1982). Avian reovirus polypeptides: analysis of intracellular virus-specified products, virions, top component, and cores. J. Virol..

[bib0017] Spandidos D.A., Graham A.F. (1976). Physical and chemical characterization of an avian reovirus. J. Virol..

[bib0018] Tang K.N., Fletcher O.J., Villegas P. (1987). Comparative study of the pathogenicity of avian reoviruses. Avian Dis..

[bib0019] Tang Y., Lu H. (2015). Genomic characterization of a broiler reovirus field strain detected in Pennsylvania. Infect. Genet. Evol..

[bib0020] Tantawi H.H., Amina N., Youssef Y.I., Fawzia M., Al-Abdulla J.M., El-Batrawi A., El-Ghawas A., Nasser A.A., Reda I.M. (1984). Infectious tenosynovitis in broilers and broiler breeders in Egypt. Vet. Res. Commun..

[bib0021] Teng L., Xie Zhixun, Xie L., Liu J., Pang Y., Deng X., Xie Zhiqin, Fan Q., Luo S., Feng J., Khan M.I. (2014). Sequencing and phylogenetic analysis of an avian reovirus genome. Virus. Genes..

[bib0022] Troxler S., Rigomier P., Bilic I., Liebhart D., Prokofieva I., Robineau B., Hess M. (2013). Identification of a new reovirus causing substantial losses in broiler production in France, despite routine vaccination of breeders. Vet. Rec..

[bib0023] Varela R., Benavente J. (1994). Protein coding assignment of avian reovirus strain S1133. J. Virol..

[bib0024] Yang J., Tian J., Chen L., Tang Y., Diao Y. (2018). Isolation and genomic characterization of a novel chicken-orign orthoreovirus causing goslings hepatitis. Vet. Microbiol..

