# Supplementary material for: Pathogenicity of avian reovirus variant in the immune organs of broiler chicks
Source: Virus Res. 2025 Feb 6;353:199538. doi: 10.1016/j.virusres.2025.199538 (PMC11848764; doi:10.1016/j.virusres.2025.199538)
Supplement: Supplementary file 1 [file mmc1.docx]

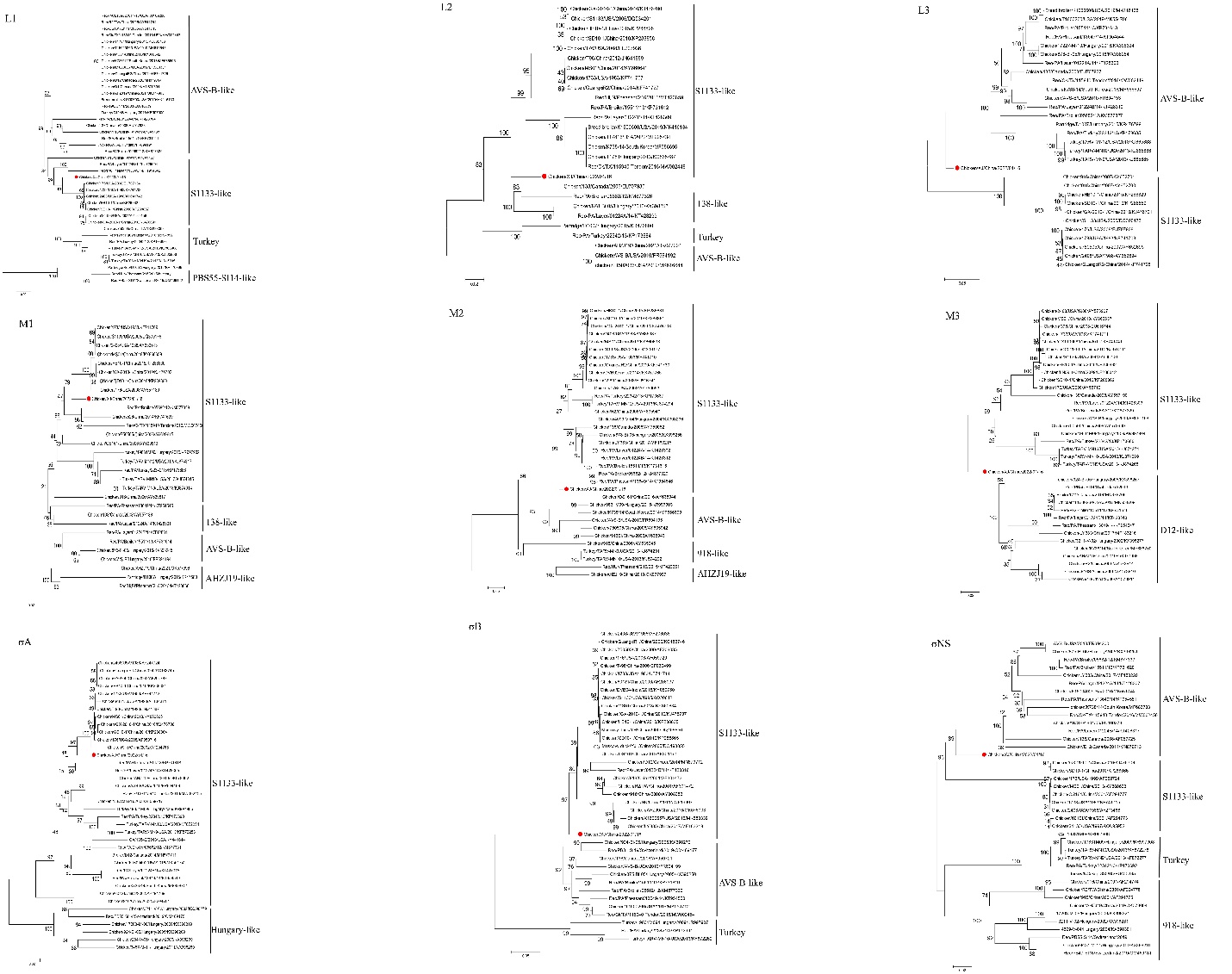


**Figure. 2-** **Supplement: Phylogenetic trees constructed by avian orthoreovirus (ARV) based on nucleotide sequences of the L-class, M-class and σ-class homologous genome segments or genes.** Note: The Chicken/XJ/China/2022/01/16strain was marked with a red-colour circle.
